# First-in-human phase I study of BPI-9016M, a dual MET/Axl inhibitor, in patients with non-small cell lung cancer

**DOI:** 10.1186/s13045-019-0834-2

**Published:** 2020-01-16

**Authors:** Xingsheng Hu, Xin Zheng, Sheng Yang, Lin Wang, Xuezhi Hao, Xinge Cui, Lieming Ding, Li Mao, Pei Hu, Yuankai Shi

**Affiliations:** 1 Department of Medical Oncology, National Cancer Center/National Clinical Research Center for Cancer/Cancer Hospital, Chinese Academy of Medical Sciences and Peking Union Medical College, Beijing Key Laboratory of Clinical Study on Anticancer Molecular Targeted Drugs, No. 17 Panjiayuan Nanli, Chaoyang District, Beijing, 100021 China; 2 Clinical Pharmacology Research Center, Peking Union Medical College Hospital, No. 41 Damucang Hutong, Xicheng District, Beijing, 100032 China; 3 Betta Pharmaceutical Co., Ltd., Hangzhou, Zhejiang China

**Keywords:** C-MET, Tyrosine kinase inhibitor, 9016-M, Phase 1, Non-small cell lung cancer (NSCLC)

## Abstract

**Background:**

BPI-9016M is a novel small-molecule inhibitor that simultaneously targets both c-Met and AXL tyrosine kinases. This phase I study aimed to determine the maximum tolerated dose (MTD), safety, pharmacokinetics, and antitumor activity of BPI-9016M in Chinese patients with advanced non-small cell lung cancer (NSCLC).

**Methods:**

Over the dose range of 100 mg to 800 mg, eligible patients were administered with a single dose of 9016M tablet and received 7 days of pharmacokinetics evaluation, followed by continuous dose administration (QD dosing, 28 days). Standard “3 + 3” dose escalations were performed.

**Results:**

Twenty NSCLC patients were treated. All patients experienced at least one adverse event (AE), of which treatment-related adverse events (TRAEs) were reported in 17 (85.0%) patients. The most common TRAEs were alanine transaminase (ALT) elevation (60%), bilirubin increased (40%), dysgeusia (40%), constipation (30%), hypertension (25%), and palmar-plantar erythrodysesthesia syndrome (15%). The TRAEs of grade 3 or higher during treatment were hypertension (15%), pulmonary embolism (5%), and laryngeal pain (5%). No dose-limiting toxicity (DLT) was observed, and the MTD was not reached. The median time to *C*_max_ ranged from 2.0 to 3.5 h, and the plasma concentration of BPI-9016M declined rapidly after *T*_max_ fitting a single-compartment model. The mean AUC_0–72 h_ of M1 and M2-2, main metabolites of BPI-9016M, were 4.8–6.6 folds and 4.1–9.8 folds higher than that of BPI-9016M, respectively. Exposure to BPI-9016M, M1, and M2-2 reached moderate saturation at 600 mg. Among 19 evaluable patients, 1 had a partial response and 10 patients had stable disease.

**Conclusion:**

BPI-9016M showed favorable safety and pharmacokinetic profiles, and no DLT was observed at doses up to 800 mg once daily. The promising antitumor activity in Chinese NSCLC patients supports further development of this tyrosine kinase inhibitor.

**Trial registration:**

Clinical Trial ID: NCT02478866, registered May 21, 2015.

## Background

c-MET is a membrane-spanning receptor tyrosine kinase encoded by the MET gene and structurally binds with hepatocyte growth factor (HGF) with high affinity [1]. Dysregulation of HGF/c-MET signaling results in activation of downstream pathways, including the RAS/MAPK, PI3K/AkT, and Rac/Rho pathways, which are involved in the cell proliferation, survival, and metastasis [2, 3]. High-level MET gene amplification, protein overexpression, or gene mutations are main mechanisms that induce aberrant activation of the HGF/c-MET pathway, and accumulating evidence has established the role of the c-Met receptor tyrosine kinase in tumor development and metastatic progression [4]. Furthermore, dysregulation of the MET tyrosine kinase is associated with resistance to targeted therapies in cancer patients and frequently occurs in non-small cell lung cancer (NSCLC) patients with EGFR inhibitor resistance. MET protein overexpression has been reported in 20–37% of tumor tissues, and MET gene amplification in 5–26% of NSCLC patients with EGFR inhibitor resistance [5–9]. Meanwhile, MET gene mutation has been reported in about 2–4% of NSCLC adenocarcinoma and in 1–2% of other NSCLC subsets [10–12]. Therefore, agents targeting MET signaling are expected to improve the treatment of this patient population with MET dysregulation. So far, a range of strategies to inhibit the HGF/MET signaling pathway has been explored. Monoclonal antibodies that directly against HGF or MET, such as onartuzumab, rilotumumab, and emibetuzumab, have entered early phase clinical trials and demonstrated promising activity [13, 14]. Several small-molecule tyrosine kinase inhibitors that inhibit a number of intracellular pathways including MET, such as cabozantinib, savolitinib, and capmatinib, are also under development [15–17].

AXL was aberrant and was involved in epithelial-mesenchymal transition (EMT) in various cancers, including NSCLC [18, 19]. Moreover, AXL overexpression was observed in 20% of NSCLC patients with resistance to EGFR inhibitors; in other cancers, enhanced AXL expression was also involved in intrinsic or acquired resistance to PI3K inhibitors, anti-HER2 treatment, immune checkpoint inhibitors in addition to chemotherapy and radiotherapy [19–22]. Recently, AXL-targeting therapies may be beneficial for NSCLC patients bearing wild-type EGFR tumors with mesenchymal features and frequent KRAS mutations, and those harboring EGFR-activating mutations with resistance to EGFR inhibitors [23].

BPI-9016M, a novel small-molecule inhibitor independently developed by Betta Pharmaceuticals Co., Ltd. (Hangzhou, China), simultaneously targets both c-Met and AXL tyrosine kinase (another transmembrane receptor tyrosine kinase, whose binding with GAS6 activates multiple downstream signaling pathways, triggering cell proliferation, angiogenesis, invasion, and metastasis). In preclinical studies, BPI-9016M could inhibit multiple kinases in vitro at 0.2 μM including the c-MET [h], AXL [h], KDR [h], DDR2[h], and Ron [h] with inhibition of 88–100%, and the half maximal inhibitory concentrations (IC_50_) of BPI-9016M against VEGFR was 30 nM, exhibiting similar pharmacological targets as cabozantinib (unpublished data). The IC_50_ values of BPI-9016M were 6 nM against wild-type c-Met tyrosine kinase and 0.12 μM against the growth of lung cancer EBC-1 cell line. M1 and M2-2 are the two main active metabolites of BPI-9016M. The IC_50_ values for M1 and M2-2 were 2 nM and 12 nM against wild-type c-Met tyrosine kinase, respectively, and 0.14 μM and 0.82 μM against the growth of the EBC-1 cell line. And the IC50 values for BPI-9016M, M1, and M2-2 against AXL kinase were 9 nM, 7 nM, and 35 nM, respectively [24]. Furthermore, promising therapeutic effects have been demonstrated on lung adenocarcinoma patient-derived xenograft (PDX) models, particularly in tumors with high expression of c-MET [25].

In the present first-in-human phase 1 study, we aimed to evaluate the maximum tolerated dose (MTD), safety, tolerability, and pharmacokinetics of BPI-9016M in patients with advanced solid tumors. Additional objective was to describe preliminary evidence of activity of BPI-9016M in this patient population.

## Patients and methods

### Study population

Eligible patients (aged 18 to 65 years) had a histologically or cytologically confirmed advanced solid tumor, for which standard therapies failed or no standard therapy was available; an Eastern Cooperative Oncology Group (ECOG) performance status of 1 or less; a life expectancy exceeding 12 weeks; an adequate hematologic, hepatic, and renal function; and a measurable disease according to Response Evaluation Criteria in Solid Tumor (RECIST) version 1.1. Eligible patients should not have received any cytotoxic chemotherapy, radiotherapy, immunotherapy, or hormone therapy within 4 weeks before study treatment. Targeted therapies should have been terminated more than 14 days or 5 half-lives of the drug (whichever was longer) before study treatment. All drug-related toxicities (except for hair loss) had to have resolved to grade 2 or lower per Common Terminology Criteria for Adverse Events (CTCAE) version 4.03. The complete lists of inclusion or exclusion criteria can be found in the Additional file 1.

### Study design

The primary objective was to establish the MTD of once daily BPI-9016M tablet in Chinese patients with advanced solid tumor. Secondary objectives included assessments of safety, tolerability, plasma pharmacokinetics (PK), and preliminary antitumor activity.

In this open-label phase 1 trial, a starting dose of 100 mg was determined based on preclinical data in rats and beagle dogs. Six dose groups were set in the dose escalation: 100, 200, 300, 450, 600, and 800 mg. In the cycle 0, patients were administered with a single dose of BPI-9016M and received 7 days of PK evaluation. Thereafter, a standard 3 + 3 dose-escalation design was used in the cycle 1 (28 days). The MTD was defined as the highest dose at which less than 33% of patients experienced a dose-limiting toxicity (DLT).

This study was performed in accordance with the Declaration of Helsinki and the principles of Good Clinical Practice. The protocol was approved by the ethics review board at each site, and all patients provided written informed consent. This study was registered with ClinicalTrials.gov (No.NCT02478866).

### Study assessments

Adverse events (AEs) were assessed and documented with the CTCAE (version 4.03) throughout the study and for 28 days after the end of the treatment. All safety analyses were undertaken in patients who received at least one dose of BPI-9016M and had safety assessments during follow-up. Tumor assessments were performed at baseline and every 8 weeks (cycle 2) until the disease progressed or intolerable adverse reactions occurred. Contrast-enhanced computerized tomography was used for scans of the chest, abdomen, and pelvis, while gadolinium-enhanced MRI used for brain scans, at screening and subsequent assessments in all patients. A consistent imaging modality was required throughout the study. The tumor responses were assessed by investigators per RECIST v1.1. Efficacy was evaluated by best overall response consisting of complete response (CR), partial response (PR), stable disease (SD), and progressive disease (PD). Hematology and biochemistry assessments were undertaken at screening and at predefined intervals during the study.

### Pharmacokinetics assessment

For the single-dose PK test in the cycle 0, serial peripheral blood samples were collected at pre-dose and at 0.5, 1, 2, 3, 4, 6, 8, 12, 16, 24, 48, 72, and 144 h post-dose. For the continuous dose PK test in the cycle 1, serial peripheral blood samples were collected at pre-dose on days 8, 15, 22, and 28, and at 0.5, 1, 2, 3, 4, 5, 6, 8, 12, 16, 24, 48, and 72 h post-dose on day 28. All serial peripheral blood samples were collected in heparin sodium anticoagulation tubes and centrifuged at 1900*g* for 10 min before storage at − 80 °C until analysis. The plasma concentration of BPI-9016M and its active metabolites were measured using a validated liquid chromatography-tandem mass spectrometry method [18].

Dose escalation was discontinued at MTD or if pharmacokinetic data (maximum plasma concentration and area under concentration-time curve) reached saturation. Patients who had CR, PR, or SD at the end of cycle 1 were permitted to continue receiving BPI-9016M tablets at the same dose. Thereafter, the safety assessments were conducted every 4 weeks, and tumor assessments were conducted every 8 weeks until disease progression or intolerable toxicity occurs.

### Statistical analysis

Safety and efficacy analyses were conducted in the full analysis set (FAS), which included patients who received at least one dose of BPI-9016M. Objective response rate (ORR) was defined as the proportion of patients with CR and PR, and disease control rate (DCR) was defined as the proportion of patients with CR, PR, and SD. Descriptive analyses of baseline status, medical history, laboratory examinations, safety indices, etc. were used to compare qualitative and quantitative data. The 95% confidence interval was calculated using approximate normal distribution method or exact probabilities method, as appropriate. The analyses were conducted by SAS 9.4 software (SAS Institute, Cary, NC, USA). PK analyses were conducted in all patients with evaluable PK concentrations using non-compartmental methods with Phoenix 8.0 (Certara, LP, Princeton, NJ, USA), and parameters included maximum observed concentration (*C*_max_), AUC, time to reach maximum plasma concentration (*T*_max_), and half-life (T_1/2_).

## Results

### Patients

Between August 2015 and November 2017, a total of 20 Chinese patients were enrolled and assigned to six groups (100 mg/qd, *n* = 4; 200 mg/qd, *n* = 3; 300 mg/qd, *n* = 3; 450 mg/qd, *n* = 4; 600 mg/qd, *n* = 3; and 800 mg/qd, *n* = 3). The basic demographic and disease characteristics of the 20 patients are presented in Table 1. All patients had non-small cell lung cancer (NSCLC), including 19 cases (95%) of adenocarcinoma and only 1 case (5%) of squamous cell carcinoma, who was assigned to the 100 mg/qd dose group. Among the 20 patients, 17 (85%) had three or more metastases at baseline and 9 (45%) had brain metastases. Prior therapies included chemotherapy and targeted therapy (100%), surgery (50%), and radiotherapy (30%). Tissues from 11 patients were tested for the c-Met mutation before treatment; 3 had a MET gene amplification confirmed by fluorescence in situ hybridization (FISH) testing and 8 had a c-MET overexpression (≥ 2+) confirmed by immunohistochemistry (IHC). No statistically significant differences in demographic data were found among the different dosing groups (Table 1). However, the biomarkers was not our main objective, so the EGFR and other driver oncogene (ALK, ROS1, RET) mutation status was not detected in this study. We just collected previous test history of patients as follows: a total of 17 patients were tested for EGFR before treatment, and numbers of patients harbored Exon 19, 20, and 21 mutation were 8, 1, and 3, respectively. Among 14 patients tested for KRAS before treatment, only one harbored codon 12 mutation in exon 2. Besides, two patients tested for B-Raf before treatment had no mutation in exon 15.

**Table 1:** Demographic and baseline characteristics

		100 mg/qd	200 mg/qd	300 mg/qd	450 mg/qd	600 mg/qd	800 mg/qd	Total
		(*n* = 4)	(*n* = 3)	(*n* = 3)	(*n* = 4)	(*n* = 3)	(*n* = 3)	(*N* = 20)
Gender	Male	1 (25.0%)	2 (66.7%)	2 (66.7%)	1 (25.0%)	2 (66.7%)	1 (33.3%)	9 (45.0%)
	Female	3 (75.0%)	1 (33.3%)	1 (33.3%)	3 (75.0%)	1 (33.3%)	2 (66.7%)	11 (55.0%)
Age (years)	Mean (SD)	47.8 (6.45)	52.0 (7.55)	54.3 (6.11)	61.3 (5.12)	55.0 (6.00)	47.0 (4.58)	53.1 (7.32)
ECOG score	0	1 (25.0%)	1 (33.3%)	1 (33.3%)	1 (25.0%)	1 (33.3%)	0	5 (25.0%)
	1	3 (75.0%)	2 (66.7%)	2 (66.7%)	3 (75.0%)	2 (66.7%)	3 (100.0%)	15 (75.0%)
Pathology	Adenocarcinoma	3 (75.0%)	3 (100.0%)	3 (100.0%)	4 (100.0%)	3 (100.0%)	3 (100.0%)	19 (95.0%)
	Squamous	1 (25.0%)	0	0	0	0	0	1 (5.0%)
Stage	IV	4 (100.0%)	3 (100.0%)	3 (100.0%)	4 (100.0%)	3 (100.0%)	3 (100.0%)	20 (100.0%)
Brain metastases	Yes	1 (25.0%)	2 (66.7%)	1 (33.3%)	2 (50.0%)	1 (33.3%)	2 (66.7%)	9 (45.0%)
	No	3 (75.0%)	1 (33.3%)	2 (66.7%)	2 (50.0%)	2 (66.7%)	1 (33.3%)	11 (55.0%)
Number of metastatic lesions	1	1 (25.0%)	0	0	0	0	0	1 (5.0%)
	2	1 (25.0%)	0	0	0	0	1 (33.3%)	2 (10.0%)
	3	1 (25.0%)	1 (33.3%)	2 (66.7%)	2 (50.0%)	2 (66.7%)	1 (33.3%)	9 (45.0%)
	4	0	0	0	1 (25.0%)	0	0	1 (5.0%)
	5	0	2 (66.7%)	1 (33.3%)	1 (25.0%)	1 (33.3%)	0	5 (25.0%)
	6	1 (25.0%)	0	0	0	0	1 (33.3%)	2 (10.0%)
Surgery	Yes	2 (50.0%)	2 (66.7%)	1 (33.3%)	2 (50.0%)	2 (66.7%)	1 (33.3%)	10 (50.0%)
	No	2 (50.0%)	1 (33.3%)	2 (66.7%)	2 (50.0%)	1 (33.3%)	2 (66.7%)	10 (50.0%)
Radiotherapy	Yes	1 (25.0%)	1 (33.3%)	0	3 (75.0%)	1 (33.3%)	0	6 (30.0%)
	No	3 (75.0%)	2 (66.7%)	3 (100.0%)	1 (25.0%)	2 (66.7%)	3 (100.0%)	14 (70.0%)
Chemotherapy and targeted therapy	Yes	4 (100.0%)	3 (100.0%)	3 (100.0%)	4 (100.0%)	3 (100.0%)	3 (100.0%)	20 (100.0%)
	No	0	0	0	0	0	0	0

### Safety

All 20 enrolled patients were included in the safety analysis set (SS), and all patients were in the relative dose intensity category of 80 to 120%. Nineteen (95%) patients experienced at least one AE, of which treatment-related AEs were reported in 17 (85%) patients. AEs reported by > 20% of patients were increased alanine transaminase (ALT) (40%), dysgeusia (40%), constipation (40%), increased conjugated bilirubin (25%), increased hemobilirubin (25%), nausea (25%), and hypertension (25%), with 9 patients (45%) experiencing grade 3 AEs. Treatment-related adverse events were predominantly grade 1 or 2, most commonly increased ALT (40%), dysgeusia (40%), constipation (30.0%), and hypertension (25%). Four patients (20%, 300 mg/qd: *n* = 1; 600 mg/qd: *n* = 2; 800 mg/qd: *n* = 1) experienced treatment-related adverse events (TRAEs) of grade 3 or higher during treatment, including hypertension (15%), pulmonary embolism (5%), and laryngeal pain (5%). AEs (any grades) leading to dose adjustment and dose discontinuation were reported in one patient (600 mg/qd group) and four patients (600 mg/qd group, *n* = 2; 800 mg/qd group, *n* = 2), respectively. One serious adverse event (pulmonary embolism, in the 600 mg/qd group) was recorded and considered probably related to study treatment. Table 2 summarizes TRAEs reported by 10% or more of patients. As no DLT was observed, the MTD was not determined.

**Table 2:** Treatment-related adverse events reported by 10% or more of patients in the safety population

	Any grade 1	Grade ≥ 3
Increased ALT	8 (40%)	0
Increased conjugated bilirubin	5 (25%)	0
Increased hemobilirubin	5 (25%)	0
Increased gamma-glutamyltransferase	4 (20%)	0
Increase AST	4 (20%)	0
Increased unconjugated bilirubin	3 (15%)	0
Increased creatine phosphokinase	3 (15%)	0
Dysgeusia	8 (40%)	0
Constipation	6 (30%)	0
Palmar-plantar erythrodysesthesia	3 (15%)	0
Papulosis	2 (10%)	0
Hypertension	5 (25%)	3 (15%)

Adverse events were assessed according to the National Cancer Institute Common Terminology Criteria version 4.0

*ALT* alanine aminotransferase, *AST* aspartate aminotransferase

### Pharmacokinetics

Pharmacokinetic analyses were performed for both the single-dose administration and continuous dose administration of BPI-9016M tablets, and all determined pharmacokinetic parameters for either the single dose or multiple doses were listed in Table 3. PK analyses after single-dose administration (100 mg to 800 mg) showed that the mean *C*_max_ ranged from 241 to 987 ng/mL, and the median time to *C*_max_ ranged from 2.0 to 3.5 h. The plasma concentration of BPI-9016M declined rapidly after *T*_max_, fitting a single-compartment model, and the mean *t*_1/2_ (half-life) ranged from 7.9 to 37.3 h. M1 and M2-2 were major active metabolites of BPI-9016M identified in preclinical study, with respective 4.8–6.6 folds and 4.1–9.8 folds of mean AUC_0–72 h_ compared to that of BPI-9016M in the current study. Over the dose range (100 to 800 mg), the plasma exposures (AUC_0–last_) of prototype BPI-9016M and M1 increased slightly less than dose proportionally, while AUC_0–last_ of M2-2 increased obviously less than dose proportionally. Additionally, maximal plasma concentration (*C*_max_) of BPI-9016M, M1, and M2-2 increased obviously less than dose proportionally. Exposure to BPI-9016M, M1, and M2-2 (AUC and *C*_max_) reached moderate saturation at 600 mg after single-dose administration, and therefore, dose escalation stopped at 800 mg.

**Table 3:** Major pharmacokinetic parameter for BPI-9016M, M1, and M2-2 in Chinese patients with advanced solid tumors after treatment with multiple doses of oral BPI-9016M tablets

		100 mg/qd	200 mg/qd	300 mg/qd	450 mg/qd	600 mg/qd	800 mg/qd
		(*n* = 4)	(*n* = 3)	(*n* = 3)	(*n* = 4)	(*n* = 3)	(*n* = 3)
*T*_max,ss_(h)	BPI-9016M	2.00(2.00–2.00)	2.00(1.00–5.00)	3.00(2.00–10.0)	3.50(2.37–6.00)	3.00(3.00–6.00)	6.00(4.00–6.00)
	M1	0.00(0.00–5.00)	6.00(0.00–8.00)	2.00(0.00–2.00)	10.0(2.37–24.0)	6.00(0.500–12.0)	3.00(0.00–4.00)
	M2-2	0.500(0.00–0.500)	1.00(0.00–24.0)	0.500(0.00–2.00)	20.0(0.500–24.0)	6.00(0.500–24.0)	1.00(0.00–24.0)
*C*_max,ss_ (ng/mL)	BPI-9016M	256(199)	571(341)	678(624)	731(150)	986(336)	963(210)
	M1	629(200)	2010(660)	2270(882)	3420(919)	3600(1540)	5770(2700)
	M2-2	563(115)	1460(155)	2520(1400)	3210(1200)	2550(1720)	4810(2390)
AUC_last_ (h*ng/mL)	BPI-9016M	1760(949)	8220(6500)	5720(5480)	11,200(3010)	14,000(5430)	17,600(6940)
	M1	17,600(5200)	99,100(57100)	69,300(55800)	161,000(59300)	147,000(63800)	261,000(124000)
	M2-2	20,700(2950)	85,800(19000)	87,800(71300)	169,000(99100)	118,000(64400)	240,000(133000)
*t*_1/2_ (h)	BPI-9016M	8.79(1.95)	21.0(11.1)	12.2(2.74)	10.5(2.23)	13.8(3.07)	11.3(2.86)
	M1	12.1(0.922)	21.7(8.11)	17.8(7.04)	20.8(2.14)	25.6(2.13)	26.2(0.694)
	M2-2	20.8(4.63)		15.9(NA)			
Ke (1/h)	BPI-9016M	0.0818(0.0197)	0.0386(0.0159)	0.0587(0.0119)	0.0684(0.0137)	0.0522(0.0122)	0.0647(0.0185)
	M1	0.0573(0.00417)	0.0344(0.0129)	0.0445(0.0216)	0.0335(0.00345)	0.0272(0.00238)	0.0265(0.000701)
	M2-2	0.0341(0.00759)		0.0437(NA)			
CLss/F (L/h)	BPI-9016M	75.0(35.0)	55.2(39.2)	106.0(77.0)	56.6(10.4)	61.8(26.9)	65.6(24.4)
Vz,ss/F (L)	BPI-9016M	963(579)	1530(872)	2030(1840)	841(165)	1150(227)	1070(499)

Values are expressed as mean (standard deviation [SD])

*T*_*max*_ is expressed as median (min–max), *T*_max,ss_ time to maximum plasma concentration at steady state, *C*_*max,ss*_ maximum plasma concentration occurring at steady state, *AUC*_*last*_ area under the time-concentration curve from the time point of first dosing to the last time point with a measurable (positive) concentration; *t*_*1/2*_ terminal time of half-life, *Ke* first order rate according to the terminal (log-linear) point of the curve, *AUCINF_pred* area under the time-concentration curve from the time of first dosing to infinity, calculated by prediction of the last observed plasma concentration, *CL/F,ss* overall body clearance at steady state for extravascular dosage, *V/F,ss* total volume of drug distribution at steady state according to the terminal phase

**Fig. 1:**
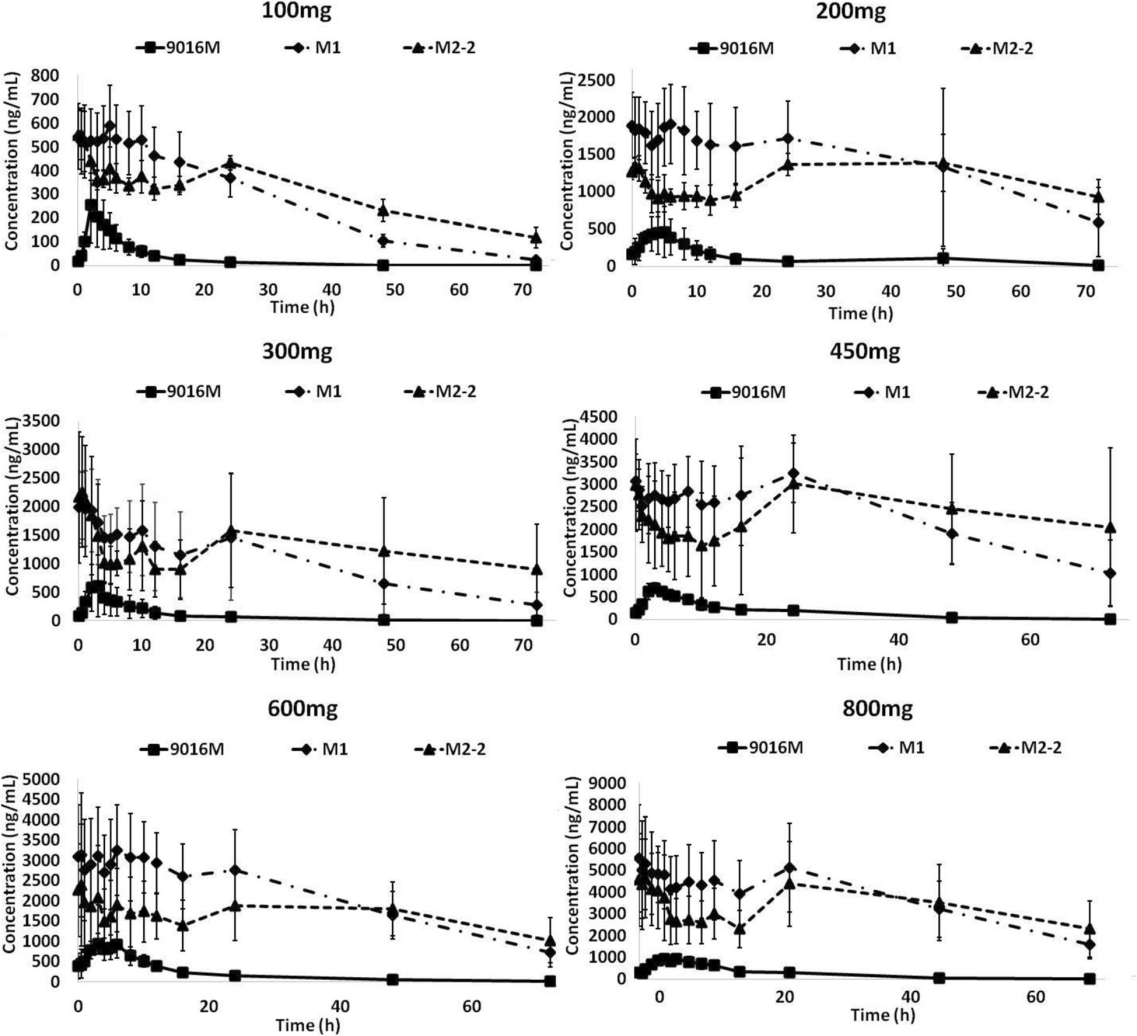
Plasma concentration-time curve ofBPI-9016M following continuous QD dosing. Average concentration-time curves for BPI-9016M, M1, and M2-2 in Chinese advanced NSCLC patients with single oral administration of 100–800 mg of BPI-9016M tablet

In continuous dose administration (QD dosing) over the dose range of 100 mg to 800 mg, a steady-state concentration of BPI-9016M was reached after 28 days. The plasma concentration-time curves of BPI-9016M following continuous dosing were shown in Fig. 1. The mean *C*_max_ (256 to 963 ng/mL), mean *T*_max_ (2.0 to 6.0 h), and *t*_1/2_ (8.8 to 21.0 h) were similar with that in single administration. No obvious accumulation of BPI-9016M was observed at steady state, with accumulation ratios ranging from 0.9 to 2.9 (compared with the AUC_0–24_ in the single-dose administration). By contrast, the accumulation ratios of M1 and M2-2 after continuous dose administration were 1.8–6.2 and 2.8–6.3, respectively. Mean steady-state plasma exposure of M1 and M2-2 were 6.4–11.0 folds and 3.6–9.4 folds higher than that of prototype BPI-9016M, respectively.

### Efficacy

**Fig. 2:**
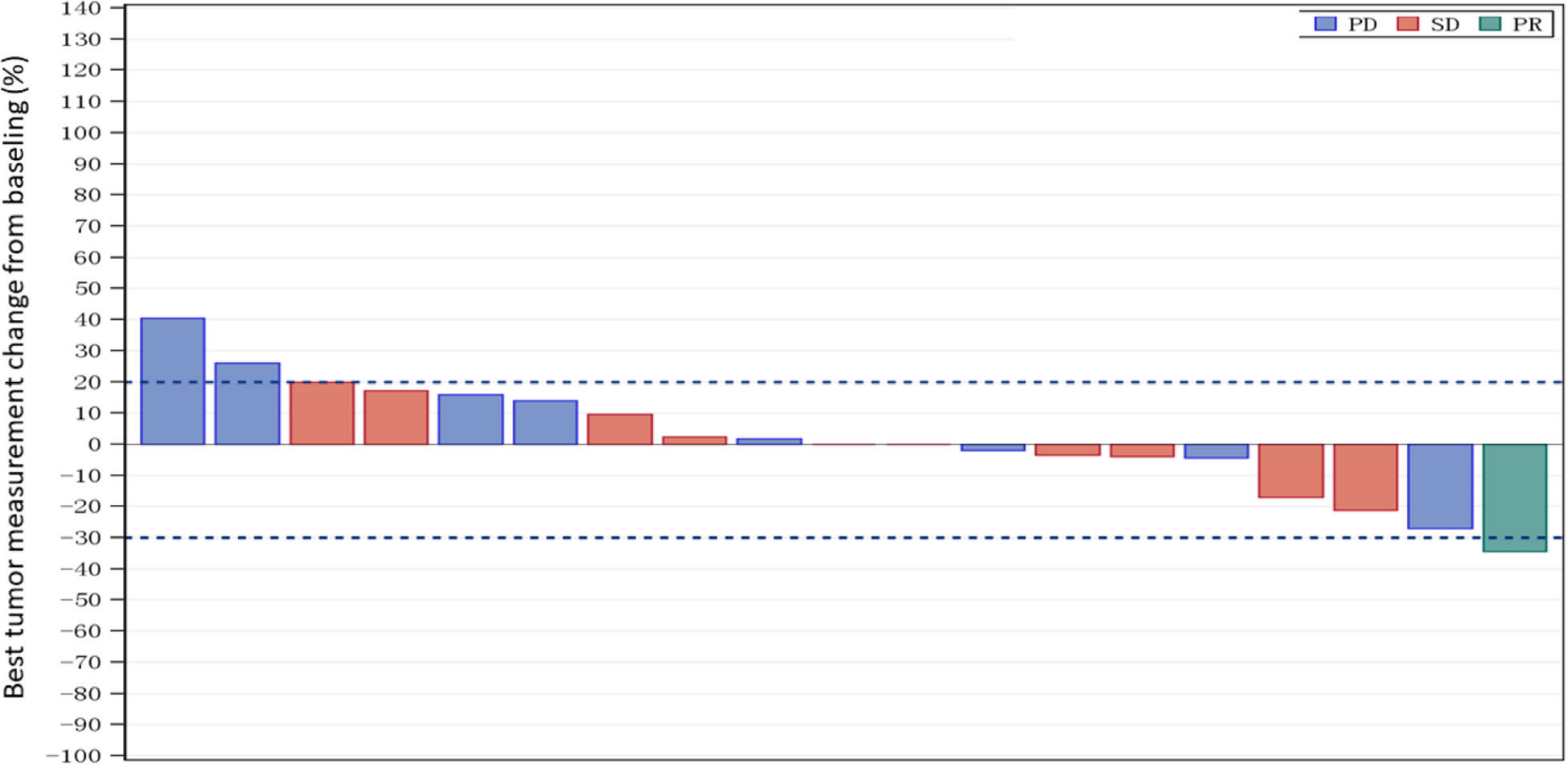
Waterfall plot of the best overall response. The bars indicate the largest percentage change in target lesions from baseline. The colors represent different best tumor response. The lower horizontal dashed line indicates a 30% reduction from baseline. The upper horizontal dashed line indicates a 20% increase from baseline

Overall, 19 patients had evaluable post-treatment tumor assessments, and tumor burden was reduced from baseline in 53% of patients (Fig. 2). One patient (in the 800 mg/qd group) displayed confirmed PR, and 10 patients had stable disease. The ORR was 5% (95% CI 0.1–26%, Table 4), and the DCR was 58% (95% CI 34–80%, Table 4). The exploratory efficacy analysis showed that among patients (*n* = 11) who had MET gene amplification or c-MET overexpression detected previously, one patient achieved PR and eight patients had SD. Thus, the ORR and DCR were 8% and 66%, respectively. By contrast, among the eight patients without MET gene amplification or c-MET overexpression, the best overall response were SD achieved by three patients.

**Table 4:** Tumor responses assessed by investigator

	Group						
	1 0 mg/qd	200 mg/qd	300 mg/qd	450 mg/qd	600 mg/qd	800 mg/qd	Total
	(*n* = 4)	(*n* = 3)	(*n* = 3)	(*n* = 4)	(*n* = 3)	(*n* = 3)	(*N* = 20)
Partial remission (PR)	0	0	0	0	0	1 (33.3%)	1 (5.3%)
Stable disease (SD)	0	2 (66.7%)	2 (66.7%)	2 (50.0%)	2 (66.7%)	2 (66.7%)	10 (52.6%)
Progressive disease (PD)	3 (100.0%)	1 (33.3%)	1 (33.3%)	2 (50.0%)	1 (33.3%)	0	8 (42.1%)
Total	3 (100.0%)	3 (100.0%)	3 (100.0%)	4 (100.0%)	3 (100.0%)	3 (100.0%)	19 (100.0%)
Missing	1	0	0	0	0	0	1
Objective remission rate, % (95%CI)	0 (NA, NA)	0 (NA, NA)	0 (NA, NA)	0 (NA, NA)	0 (NA, NA)	33.3 (0.84, 90.57)	5.3 (0.13, 26.03)
Disease control rate, % (95%CI)	0 (NA, NA)	66.7 (9.43, 99.16)	66.7 (9.43, 99.16)	50.0 (6.76, 93.24)	66.7 (9.43, 99.16)	100.0(29.24,100.0)	57.9 (33.50, 79.75)

The objective remission rate (ORR) and disease control rate (DCR) were calculated based on the overall assessment. The ORR was calculated as the proportion of patients who achieved complete remission (CR) and partial remission (PR). The DCR was calculated as the proportion of patients who achieved CR, PR, and stable disease (SD). Overall evaluation did not require confirmation of the efficacy of CR or PR and was the best response at all points in the trial

## Discussion

This first-in-human phase I dose-escalation study demonstrated that BPI-9016M was generally well tolerated in patients with advanced NSCLC who had progressed on standard therapy or for whom no effective therapy was available. The MTD was not reached, with a maximum dose investigated being 800 mg QD.

Overall, BPI-9016M was well tolerated, indicated by the low rates of dose reduction (5%) due to AEs and by low frequency of AEs. Three patients (15%) experienced drug discontinuation due to treatment-related AE (two patients in 600 mg/qd group and one in 800 mg/qd group). In 600 mg/qd group, one patient stopped the drug treatment due to pulmonary embolism, increased fibrin d-dimer and fibrin degradation; dose interruption was seen in the other one who stopped the drug because of grade 3 hypertension, and then the patient stopped discontinued the drug by himself due to throat pain, hyperhidrosis, and abnormal feeling. In the 800 mg/qd group, patient suffered from elevated alanine aminotransferase and wheezing and suffocation due to worsened pericardial effusion. Taken these findings together, BPI-9016M was discontinued by the study physician. A previous trial reported that 12 patients (18.8%) experienced an AE leading to drug discontinuation in NSCLC patients treated with cabozantinib plus erlotinib [26]. Moreover, a phase 1 study of cabozantinib in Japanese NSCLC patients demonstrated that the rate of discontinuation due to an AE was 10% [27]. In a phase Ib/II study of capmatinib plus gefitinib in patients with EGFR-mutated, MET-dysregulated NSCLC, 27 of 161 patients (17%) reported AEs that led to study drug discontinuation [28]. A phase I dose-escalation study of capmatinibin Japanese patients with advanced solid tumors reported that 21 patients (47.7%) experienced one or more dose interruptions during the study, with the dose interruption only once in nine patients (20.5%) [16]. As a result, considering the small sample size, we believed that the rate of discontinuation due to an AE in this study was acceptable. Moreover, the AEs reported in this study were predominately grade 1 or 2. Such safety profile compared favorably with those of other MET inhibitors (cabozantinib, capmatinib, and savolitinib). For cabozantinib, a multi-kinase inhibitor with targets including MET and Axl, dose reduction and discontinuation in the dose-escalation cohorts were reported in about 30–40% and 13% of patients, respectively [27]. Furthermore, 70% of patients experienced ≥ grade 3 AEs, and the most common AEs were palmar-plantar erythrodysesthesia (100%), increased ALT (95%), increased AST (95%), hypertension (87%), and diarrhea (78%) [27]. In addition, cabozantinib alone or combination therapy also showed more grade 3 or worse AEs than erlotinib alone did [29], and cabozantinib needed to be reduced to 40 mg daily in combination with erlotinib to limit diarrhea [26], indicating that cabozantinib may be potentially less tolerable. Although increased ALT, hypertension and palmar-plantar erythrodysesthesia were also observed in our study, but the frequency was much less than that with cabozantinib. For INC280 (capmatinib), a specific c-MET inhibitor, adverse events leading to dose adjustment and/or interruption were reported in 59% of patients, while AEs leading to discontinuation were reported in 9% of patients in a recently published phase I dose-escalation study [16]. The most common AEs were increased blood creatinine (52%), nausea (48), decreased appetite (41%), vomiting (39%), and diarrhea (25%). The gastrointestinal symptoms, such as vomiting and diarrhea, were also observed frequently with savolitinib [17], while less reported (≤ 10%) with BIP-9016M. Furthermore, the ATTENTION trial was terminated because of an increased incidence of interstitial lung disease in the tivantinib group [30]. Therefore, BIP-9016M showed a distinctive profile of AEs compared with other c-MET inhibitor mentioned above, and the different specificities of these agents for c-MET may help explain the differences, but further studies are needed to investigate the underline mechanisms.

Pharmacokinetic analyses showed that the exposure to BPI-9016M and the metabolites (M1 and M2-2) reached slight saturation at 600 mg following single-dose administration, and it was considered unnecessary to proceed with further dose escalation after 800 mg. In continuous dose administration (QD dosing) over the dose range of 100 mg to 800 mg, a steady-state concentration of BPI-9016M was reached after 28 days and no obvious accumulation was observed, which was consistent with the relatively short plasma *t*_1/2_ of BPI-9016M. In comparison, the M1 and M2-2 showed a relative longer plasma *t*_1/2_, and moderate accumulation of M1 and M2-2 were observed. As the exposure (AUC) of M1 and M2-2 were about 5–10 times than that of the parent compound; therefore, it is estimated that BPI-9016M may contribute to less efficacy than its metabolites. Following continuous administration of different doses, the range of minimal plasma concentration of M1 and M2-2 at steady-state were 368–3930 ng/mL and 316–2300 ng/mL, respectively. These exposures far surpassed the IC50 in the enzymatic assays (The IC50s of BPI-9016M, M1, and M2-2 against wild-type c-Met kinase were 6 nM, 2 nM, and 12 nM, respectively) and might provide the inhibition of c-MET required to achieve favorable efficacy [24].

Preclinical study showed that BPI-9016M inhibits the phosphorylation of c-MET and its downstream signaling targets, such as ERK and AKT, in both patient-derived xenograft tumors and various MET-amplified cell lines in a dose-dependent manner, which subsequently induce cell proliferation and cell cycle progression [24]. In the present study, patients assigned in the higher dose groups tended to derive more benefits than those in lower dose groups. As this study was designed to evaluate safety and tolerability profile of BPI-9016M and a limited number of patients were enrolled, the relationship between drug exposure and clinical response should be interpreted with caution. Additionally, our preclinical study suggested that BPI-9016M significantly inhibited tumor growth of lung adenocarcinoma with overexpression of c-MET, but not of those without c-MET overexpression. Moreover, PR was the best overall response reported in one (5.2%) patient with EGFR mutation. Recently, MET inhibitors combined with EGFR-TKIs have been studied clinically in NSCLC patients, because NSCLC patients may become resistant to EGFR inhibitors due to secondary EGFR mutations, MET amplification, or HGF overexpression. However, the combination strategies failed in unselected populations but had positive results in patients with MET amplification or overexpression. The MARQUEE study showed no significantly improved overall survival, but subgroup analyses demonstrated that tivantinib plus erlotinib improved OS in patients with MET overexpression [31]. Tepotinib and gefitinib combination significantly improved response rate in patients with MET amplification or overexpression [32]. Capmatinib has also been evaluated in combination with gefitinib in patients with EGFR-mutant NSCLC who progressed on previous gefitinib treatment, the overall ORR was 27%, while strong antitumor activities were seen in patients with high MET-amplified tumors (ORR47%) [28]. Crizotinib, a dual c-MET and ALK inhibitor, has been studied in METex14-altered NSCLC patients with an unconfirmed response rate of up to 44% [33]. Another example was tepotinib (EMD 1214063), which showed promising activity in NSCLC patients harboring c-MET exon14 skipping mutations with an ORR of 60% (9/15) [34], while cabozantinib alone or combined with erlotinib has superior efficacy to that of erlotinib alone in patients with wild-type EGFR NSCLC [29]. Additionally, cabozantinib is also known as an AXL inhibitor, and biomarkers may be identified in an ongoing clinical trial in NSCLC patients with AXL overexpression, amplication, or mutation (NCT01639508). Failures of several MET inhibitors in phase III trials underscore the importance of identifying biomarkers that reliably predict benefit from MET inhibition.

Although in this phase I study, the patients had not been selected based on MET status, an exploratory analysis was performed to assess the relationship between MET status and clinical response using previous documented testing for MET dysregulation. Patients with MET gene amplification or c-MET overexpression demonstrated higher ORR (9%) and DCR (82%) compared to those without MET dysregulation (ORR, 0%; DCR, 25%). However, it should be noted that this dose-escalation phase I study could only provide preliminary estimate of antitumor activity, future studies are warranted to further evaluate the efficacy of BPI-9016M. Based on the results of this phase I dose-escalation study, we designed a phase Ib dose-expansion trial enrolling only patients with c-MET overexpression tumors (NCT: 02929290). This phase Ib study is ongoing now and will be reported in due course.

## Conclusions

In conclusion, this study suggested that orally administered BPI-9016M was well tolerated, with a favorable safety profile. The MTD was not reached, and no DLT was observed at doses up to 800 mg qd. Modest antitumor activity was observed in this dose-escalation study of patients with heavily pretreated advanced NSCLC that was not selected by MET status, adding to the clinical evidence of efficacy for BPI-9016M in NSCLC. These findings support further clinical development of BPI-9016M.

## Supplementary information

**Additional file 1.** Exclusion and inclusion criteria

## Data Availability

The datasets used and/or analyzed during the current study are available from the corresponding author on reasonable request.

## Abbreviations

AE: Adverse event
ALT: Alanine transaminase
*C*_max_: Maximum observed concentration
CR: Complete response
DCR: Disease control rate
DLT: Dose-limiting toxicity
FAS: Full analysis set
FISH: Fluorescence in situ hybridization
HGF: Hepatocyte growth factor
IC_50_: Half maximal inhibitory concentration
IHC: Immunohistochemistry
MTD: Maximum tolerated dose
NSCLC: Non-small cell lung cancer
ORR: Objective response rate
PD: Progressive disease
PDX: Patient-derived xenografts
PK: Pharmacokinetics
PR: Partial response
SD: Stable disease
SS: Safety analysis set
T_1/2_: Half-life
*T*_max_: Time to reach maximum plasma concentration
TRAEs: Treatment-related adverse events
