# Supplementary material for: First-in-human phase I study of BPI-9016M, a dual MET/Axl inhibitor, in patients with non-small cell lung cancer
Source: J Hematol Oncol. 2020 Jan 16;13:6. doi: 10.1186/s13045-019-0834-2 (PMC6966871; doi:10.1186/s13045-019-0834-2)
Supplement: Supplementary file 1 — Additional file 1. Exclusion and inclusion criteria [file 13045_2019_834_MOESM1_ESM.docx]

# Additional file 1

Inclusion criteria:

1. Patients had histologically or cytologically confirmed advanced solid tumor, for which standard therapies failed or no standard therapy was available
2. Patients had a life expectancy exceeding 12 weeks;
3. Patients were18 to 65 years
4. Patients had measurable disease according to Response Evaluation Criteria in Solid Tumor (RECIST) version 1.1.

- at least one measurable lesion. If there is only one lesion, the nature of new organisms at the site of the lesion must be histologically or cytologically confirmed.
- at least one diameter of the lesion can be accurately measured by any of the following methods: chest or abdominal computed tomography (CT) or magnetic resonance imaging (MRI), conventional method diameter (> 20 mm) or spiral CT diameter (> 10 mm).

1. Patients were of an Eastern Cooperative Oncology Group (ECOG) performance status of 1 or less;
2. Patients did not receive any cytotoxic chemotherapy, radiotherapy, immunotherapy, or hormone therapy within 4 weeks before study treatment, and recovering from all drug-related toxicities (except for hair loss) to grade 2 or lower per Common Terminology Criteria for Adverse Events (CTCAE) version 4.03.
3. Patients had targeted therapies terminated more than 14 days or 5 half-lives of the drug (whichever was longer) before study treatment.
4. Patients had adequate hematologic, hepatic, and renal function: absolute neutrophil count (ANC) ≥1.5×10^9^/L (1500/mm^3^), platelet count ≥100×10^9^/L, hemoglobin level ≥9 g/dL; prothrombin time (PT) according to the international standardized ratio or partial thromboplastin time <1.5 times the upper limit of normal (ULN); aspartate aminotransferase (AST) and alanine aminotransferase (ALT) levels ≤1.5 times the ULN (≤2.5 times ULN in cases of liver metastasis), serum creatinine level ≤5 times normal value, and endogenous creatinine clearance ≥60 mL/min.
5. Pre-menopausal women of child-bearing age had to have a negative pregnancy test within 7 days before starting treatment, and pregnant or lactating women were excluded All patients (male and female) were required to take adequate contraceptive measures throughout the treatment period and for 3 months after the end of treatment.
6. Patients signed written informed consent, and followed the therapy plan and follow-up plan.

Exclusion criteria:

1. Patients had any severe allergies to the drug or any of its excipients (microcrystalline cellulose, lactose, sodium cross-linked carboxymethyl cellulose, hydroxypropyl cellulose, magnesium stearate) were excluded.
2. Patients received any drugs avoided in this clinical study included other HGF/c-Met small molecule inhibitors or HGF/c-Met antibody drugs including crizotinib, cabozantinib, volitinib, metatinib, capmatinib, etc.
3. Patients had cerebral/meningeal metastases (except patients without hormone maintenance therapy after local treatment of brain metastases and patients with asymptomatic brain metastases); past interstitial lung disease, drug-induced interstitial disease, radiopneumonia requiring hormone therapy, any clinically evident active interstitial lung disease, or idiopathic pulmonary fibrosis on CT at baseline; uncontrolled pleural or pericardial effusions; severe or uncontrollable systemic diseases, such as unstable or uncompensated respiratory, cardiac, liver or kidney diseases, according to the investigators; and any unstable systemic disease, including any symptom greater than grade 2 on the CTCAE, uncontrollable hypertension, unstable angina, congestive heart failure, and severe liver and kidney or metabolic diseases; active hepatitis virus or human immunodeficiency virus (HIV) infection; definite history of a neurological or mental disorder, including epilepsy or dementia; history of organ transplantation; and history of major surgery or severe trauma within 4 weeks before the first treatment.
4. Patients had known or high risk of thrombosis.
5. Patients had any difficulties with drug ingestion, drug absorption or pharmacokinetic parameters of the test drug, including any type of gastrointestinal resection or surgical history.
6. Pregnant or lactating women.
7. Patients had any interferes or influences with the participation of research or the evaluation of research results, such as drug abuse and medical, psychological or social reasons.
8. Patients had any unstable or potentially influences on patient safety and patient compliance.
9. Patients had other antineoplastic treatments at the same time.
10. Patients had herbal medicines for anti-tumor effects or induction/inhibition of CYP3A4 within 7 days before the first administration.
11. Patients were not suitable for current therapy based on the investigators’ evaluation.
